# Ammonia transport mediated by urea transporter A isoforms

**DOI:** 10.1242/bio.061655

**Published:** 2025-06-06

**Authors:** N. Pina-Lopes, J. Kabutomori, R. Campos, R. Musa-Aziz

**Affiliations:** Department of Physiology and Biophysics, Institute of Biomedical Sciences, University of São Paulo, São Paulo 05508-900, Brazil

**Keywords:** *Lithobates oocytes*, Urea transporter, Membrane permeability, Water transport, Ammonia transport, Urinary concentration, Renal physiology, Renal function, Site-directed mutagenesis, Surface expression, Surface pH, Protein structure/function

## Abstract

Urea transporters (UTs) are a family of urea-selective channel proteins that play an essential role in the urine-concentrating mechanism of the mammalian kidney. In addition to urea, UT-A2 and UT-A3 – the N- and C-terminal regions of full-length UT-A1, respectively – and UT-B transport water, and human UT-B transports water and ammonia (NH_3_). However, UT-A-mediated NH_3_ transport has not been evaluated. Given that regulated renal NH_3_/NH_4_^+^ transport by renal epithelial cells is essential to acid–base homeostasis and considering UT-A2 and UT-A3 localization in the inner medulla, where the transport of urea, water, and NH_3_ is important, it is plausible that UT-A-mediated NH_3_ transport could be physiologically relevant. The present study characterized the urea, water, and NH_3_ transport properties and solute pathways of murine UT-A2, UT-A3, and UT-B heterologously expressed in *Lithobates catesbeianus* oocytes.

Control and UT-expressing oocytes were evaluated for surface protein expression through lysine-biotinylation and immunoblotting. Urea uptake was measured using radiolabeled urea, water permeability was assessed using video microscopy, and NH_3_ transport was monitored using a surface pH microelectrode.

All UT-encoding cRNAs were translated, glycosylated, and inserted into the oocyte membrane. Wild-type UT-expressing oocytes displayed significantly higher urea, water, and NH_3_ transport than day-matched water-injected control cells. Pre-treating the oocytes with phloretin or mutating the urea pore threonines (Thr177 and Thr339 human UT-B numbering) to valines (Val) attenuated UT-mediated urea, water and NH_3_ transport to control oocyte values.

Our study showed for the first time that UT-A2 and UT-A3 increase the membrane NH_3_ permeability. Thus, besides the critical role of UTs in urinary concentration, these proteins may also impact acid–base homeostasis and contribute to other processes associated with health and disease.

## Teaser

**Summary:** The central message of this work is that UT-A2 and UT-A3 function as ammonia channels and employ a transport mechanism similar to UT-B.

## INTRODUCTION

Urea transporters (UTs) are transmembrane glycoproteins that, in addition to serving as a way to excrete nitrogen, facilitate the diffusion of urea across cell membranes and play a crucial role in the urinary concentrating mechanism, an important process for maintaining nearly constant blood plasma osmolality. These transporters are solute carrier proteins encoded by two distinct genes, *SLC14A2* and *SLC14A1*, with alternative splicing producing six *SLC14A2* isoforms (UT-A1–6) and two *SLC14A1* isoforms (UT-B1–2) (Bagnasco et al., 2001; Nakayama et al., 2001; Smith, 2009). In the kidney, UT-A1 and UT-A3 are expressed primarily in the inner medullary collecting duct (IMCD), UT-A2 in the thin descending limb (tDL), and UT-B in the vasa recta. The role of these UTs in urinary concentration has been exemplified in global and targeted UT knockout (KO) mice (Fenton, 2008). Except for the UT-A2 KO animal models, which require a low-protein diet challenge to reveal their physiological role, UT KO mice cannot properly concentrate urine, consequently producing larger volumes of dilute urine (Bankir et al., 2004; Fenton, 2008; Fenton et al., 2004; Lei et al., 2011; Uchida et al., 2005; Yang and Bankir, 2005; Yang and Verkman, 2002; Yang et al., 2002).

The production of concentrated urine depends on establishing an osmotic gradient due to the transport of urea [and NaCl reabsorbed in the thick ascending limb of the Loop of Henle via Na^+^-K^+^-2Cl^−^ cotransporters (NKCC2) (Ares et al., 2011; Gamba et al., 1994; Payne and Forbush, 1994)] into the inner medulla. The urea transport process begins within the IMCD, where UT-A1 and UT-A3 on the apical membrane (Shayakul et al., 1996; Terris et al., 2001) facilitate urea reabsorption from the tubular lumen into the cells, while UT-A3 and perhaps aquaporin 3 (AQP3) on the basolateral membrane (Ishibashi et al., 1997) transport the urea back into the medullary interstitium. Importantly, since UT-A1 and UT-A3 are not physically associated, it is plausible that they are differentially regulated and may have different functions. Simultaneously, UT-B expressed in the vasa recta facilitates the bidirectional movement of urea between the blood and the medullary interstitium, while UT-A2 in the tDL of the loop of Henle facilitates the uptake of urea from the medullary interstitium into the tubular lumen, which minimizes the washout of the medullary osmotic gradient and supports the concentration of urine in a process known as urea recycling. In response to increased blood osmolality and/or low blood volume leading to low blood pressure, antidiuretic hormone (ADH) secreted by the pituitary gland upregulates AQP2 (Knepper, 1997), UT-A2 (Wade et al., 2000), UT-A1 and UT-A3 (Stewart, 2011) to maintain the hypertonic environment necessary for maintaining the osmotic gradient to drive water reabsorption.

Besides maintaining the blood plasma osmolality, the kidneys also play a critical role in maintaining acid–base homeostasis via reabsorption of virtually all the filtered bicarbonate (HCO_3_^−^) and generation of ‘new HCO_3_^−^’ to replace that consumed during the titration of the daily produced fixed acids (H^+^). The daily acid load is a measure of fixed acids produced by endogenous metabolism, fixed acids from modern western diets (rich in animal protein and poor in fruit and vegetables), and base lost in the feces. The primary mechanism of ‘new HCO_3_^−^’ generation involves the renal production and excretion of NH_4_^+^ regulated according to the acid–base state (Weiner and Verlander, 2013). Total ammonia exists both as ammonia (NH_3_) and its protonated to form ammonium (NH_4_^+^). Here, we use the term NH_3_/NH_4_^+^ to refer to the combination of both molecular forms. When referring to a specific molecular form, we state either NH_3_ or NH_4_^+^. For each NH_4_^+^ excreted, one ‘new HCO3^−^’ is generated and delivered to the bloodstream. The renal excretion of NH_4_^+^ involves integrated responses of three nephron segments of the kidney, which include specific transport mechanisms in the proximal tubule (PT), the thick ascending limb (TAL) of the loop of Henle, and the collecting duct (CD).

The NH_4_^+^ excreted in the urine is produced predominantly from glutamine in the PT cells (Weiner and Verlander, 2013), secreted into the lumen of this segment, reabsorbed in the TAL of the loop of Henle – leading to the accumulation of NH_3_/NH_4_^+^ in the interstitial fluid of the renal medulla – and then secreted by the CD from the renal interstitium into the luminal fluid for excretion in the urine. It is well accepted that ammonia (NH_3_) secretion (and parallel secretion of H^+^) by the CD cells plays an important role in NH_4_^+^ excretion and, thus, in controlling systemic pH. Indeed, increases in renal NH_4_^+^ excretion are associated with substantial increases in NH_3_/NH_4_^+^ secretion by the CD. However, the previous paradigm that NH_3_/NH_4_^+^ transport through the CD epithelium could be explained by passive NH_3_ diffusion has been challenged by the recognition that specific membrane proteins mediate the transport of NH_3_ or NH_4_^+^.

For example, earlier studies have mainly ascribed NH_3_/NH_4_^+^ transport in the outer medullary collecting duct (OMCD) to the Rhesus (Rh) glycoproteins RhBG and RhCG (Biver et al., 2008; Eladari et al., 2002; Gruswitz et al., 2010; Verlander et al., 2003; Weiner and Verlander, 2011). In contrast, much less is known about NH_3_/NH_4_^+^ handling in the inner medulla, particularly in the IMCD, and the transporters involved. One study reported that hUT-B, expressed in the vasa recta, red blood cells (RBCs), liver, and other locations throughout the body, is permeable to urea, water and NH_3_ (Geyer et al., 2013b). While UT-A2 and UT-A3 have been shown to increase the membrane permeability to urea and water, it remains unclear if and how these isoforms transport this weak base, which could shed light on the NH_3_/NH_4_^+^ handling in the inner medulla.

**Fig. 1.**
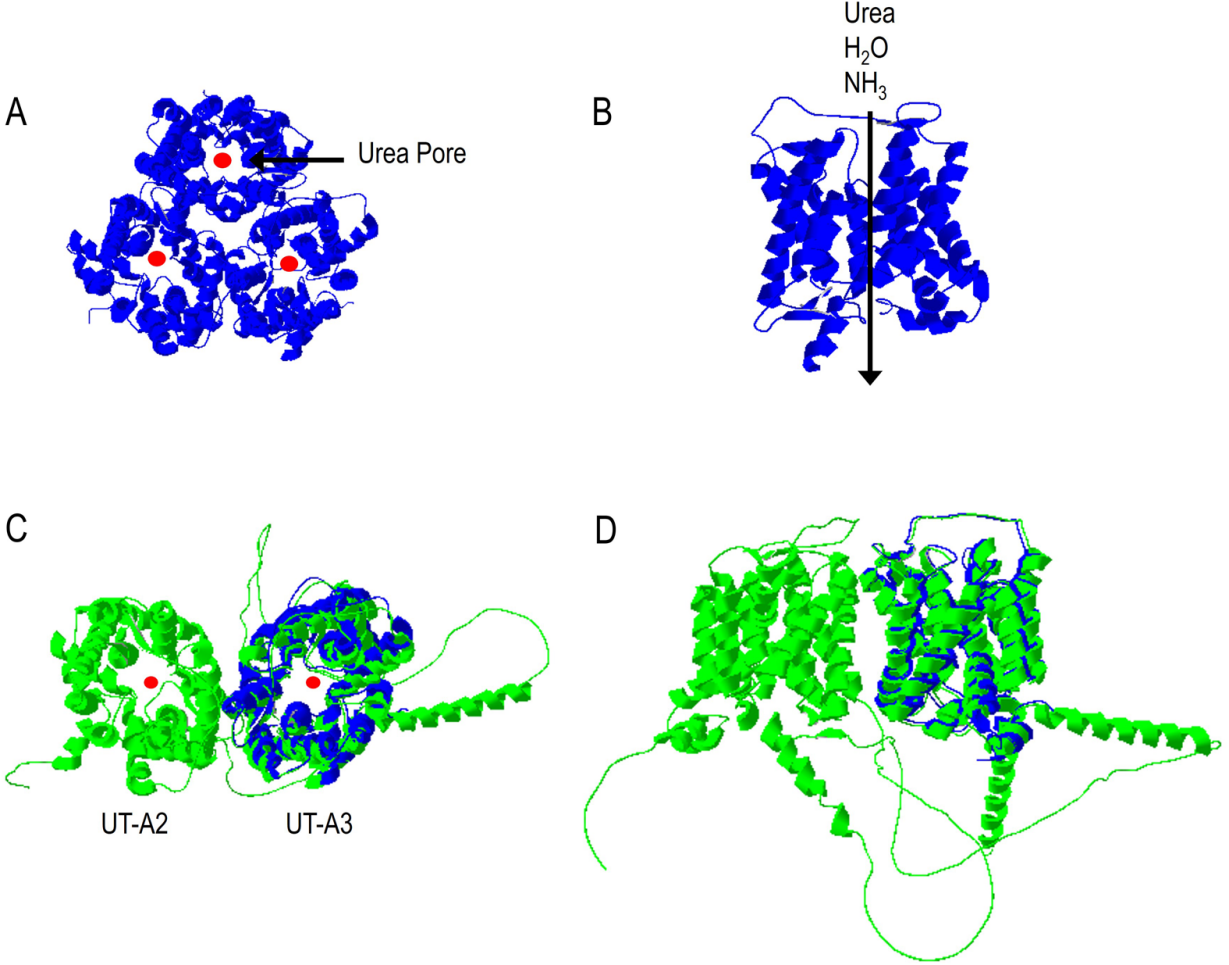
**Three-dimensional structure of human UT-B (blue) and UT-A1 (green).** (A) UT-B homotrimer (top view), (B) UT-B monomer (side view), (C) UT-A1 with UT-B superimposed on the C-terminal domain (i.e. UT-A2) (top view), and (D) UT-A1 with UT-B superimposed on the C-terminal domain (i.e. UT-A2) (side view). The structure of bUT-B (PDB ID 6QD5) was superimposed onto UT-A1 [UniProtQ15849 AlphaFold (Jumper et al., 2021; Varadi et al., 2022)] using the alignment tool in the Swiss Protein Databank Viewer program.

Crystallographic studies of a bacterial UT-B homolog (Levin et al., 2009), bovine UT-B (bUT-B) (Levin et al., 2012) and hUT-A3 (Chi et al., 2023) have revealed that each protein monomer contains ten transmembrane helices that fold to form a narrow hydrophobic urea channel and that the monomers assemble into homotrimers (Fig. 1A and B). The urea channel can be divided into three distinct regions: S_o_ (outer, extracellular), S_i_ (inner, intracellular), and S_m_ (middle, selectivity filter) (Levin et al., 2012, 2009). Site-directed mutagenesis studies of key threonine (Thr) residues in the S_m_ region (Thr177 and Thr339 in human UT-B, Thr172 and Thr334 in bovine UT-B, Thr172 and Thr334 in murine UT-B, Thr176 and Thr338 in murine UT-A2, and Thr246 and Thr408 in murine UT-A3), chemical inhibition using phloretin, and molecular dynamics experiments with mammalian UT-Bs all indicate that urea, water and NH_3_ share a common pathway (i.e. the urea channel) (Geyer et al., 2013b; Levin et al., 2012).

In contrast to UT-B, the predicted three-dimensional structure of full-length UT-A1 (929 amino acid protein) comprises two urea channel domains, which correspond to the UT-A2 (C-terminal) and UT-A3 (N-terminal) isoforms, connected by a long intracellular loop (Fig. 1C and D) (Jumper et al., 2021; Sanguinetti et al., 2022; Varadi et al., 2022). As shown in Fig. 1C and D, a bUT-B monomer can be superimposed onto the C-terminal domain of UT-A1 (i.e. UT-A2) (Fig. 1C and D). A similar fit was obtained with bUT-B and the UT-A3 N-terminal domain (not shown). While the UT-A isoforms are assumed to share the same transport properties and mechanisms, this has not been evaluated experimentally. Indeed, some protein families display different solute specificities, as evidenced by the AQP family (Geyer et al., 2013a).

Given the lack of information about NH_3_/NH_4_^+^ handling in the inner medulla and UT-A-mediated NH_3_ transport, the present study evaluated the urea, water, and NH_3_ transport properties and their pathways of UT-A2 and UT-A3 by heterologously expressing these transporters in *Lithobates catesbeianus* oocytes. We also assessed the effects of mutating the Thr residues in the urea pore's selectivity filter to evaluate the transport pathways through the UT-A isoforms. The results provide insights into renal physiology, the mechanisms underlying urinary concentration and acid–base handling in the inner medulla, and might have implications for various physiological and pathological conditions and therapies.

## RESULTS

### Surface expression

**Fig. 2.**
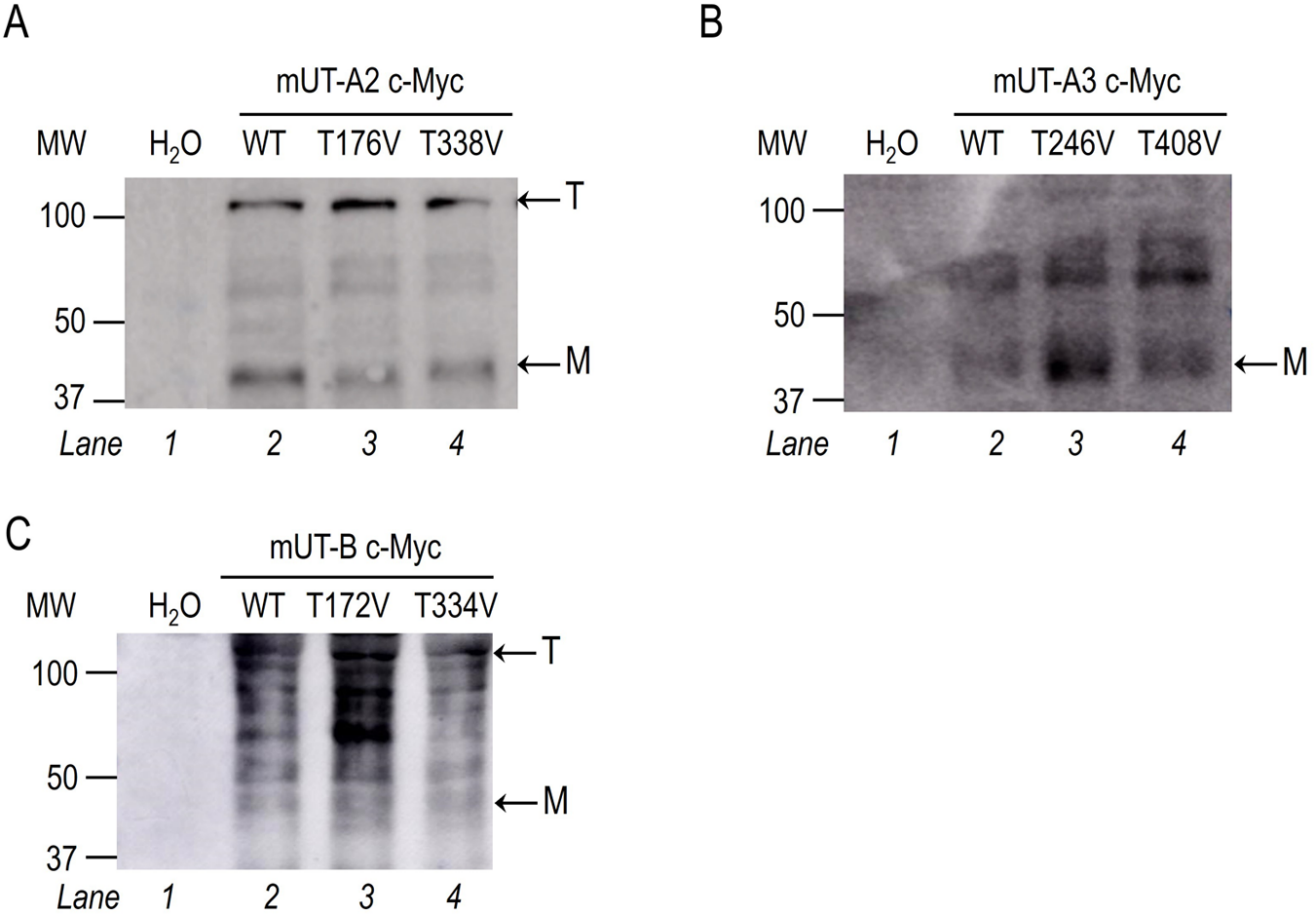
**Surface expression of wild-type and selectivity filter mutants of mUTs in *Lithobates* oocytes.** Immunoblots of biotinylated samples from oocytes injected with cRNA encoding for (A) Lane 1, H_2_O-injected controls; Lane 2, wild-type mUT-A2; Lane 3, mUT-A2^T176V^ and Lane 4, mUT-A2^T338V^. (B) Lane 1, H_2_O-injected controls; Lane 2, wild-type mUT-A3; Lane 3, mUT-A3^T246V^ and Lane 4, mUT-A3T408V. (C) Lane 1, H_2_O-injected controls; Lane 2, wild-type mUT-B; Lane 3, mUT-B^T172V^ and Lane 4, mUT-B^T334V^. A monoclonal anti-cMyc-tag antibody was used to detect the UTs. Based on each UT isoform's primary sequence, the molecular weights of each tagged monomer were expected to run at approximately 34 kDa and 68 and 102 kDa for the dimers and trimers interspersed with glycosylated forms of the proteins. The representative blots for each UT and mutants demonstrate that these membrane proteins were heterologously expressed and inserted into the cell membrane. Biotinylated H_2_O-injected controls displayed no immunoreactivity in this region. *N*=4 independent oocyte preparations and injections. T, trimer; M, monomer.

In Fig. 2A, representative immunoblots of UT-A2^WT^ (Lane 2), UT-A2^T176V^ (Lane 3), and UT-A2^T338V^ (Lane 4) surface fraction samples presented various bands (Fig. 2A). The heavier immunoreactive bands above 100 kDa correspond to the trimeric form. The other less intense bands from 50–100 kDa are likely glycosylated UT-A2 monomers and dimers. The lower molecular weight (MW) band, around 37 kDa, was previously assigned as an unglycosylated monomer (Wade et al., 2000). UT-A3^WT^ (Lane 2), UT-A3^T246V^ (Lane 3), and UT-A3^T408V^ (Lane 4) were also surface expressed, as evidenced by immunoreactive bands at ∼40 kDa, the MW of glycosylated monomeric UT-A3 (Fig. 2B). Other higher MW species (55–90 kDa) were also detected and probably correspond to glycosylated UT-A3 monomers, dimers, and/or trimers. Lastly, UT-B^WT^ (Lane 2), UT-B^T172V^ (Lane 3), and UT-B^T334V^ (Lane 4) surface expression analyses revealed a wide range of immunoreactive bands (Fig. 2C). The detected bands corresponded to glycosylated monomers (∼45–65 kDa) and trimers (∼120 kDa). Thus, the wild-type and mutant UTs were expressed on the oocyte membrane. Neither Thr mutation in any UT isoform compromised the transcription, glycosylation, or membrane insertion despite differences in immunoreactive band patterns. Moreover, none of the immunoreactive UT bands were observed in the day-matched water-injected control oocytes (Lane 1, Fig. 2A–C).

### Urea uptake

**Fig. 3.**
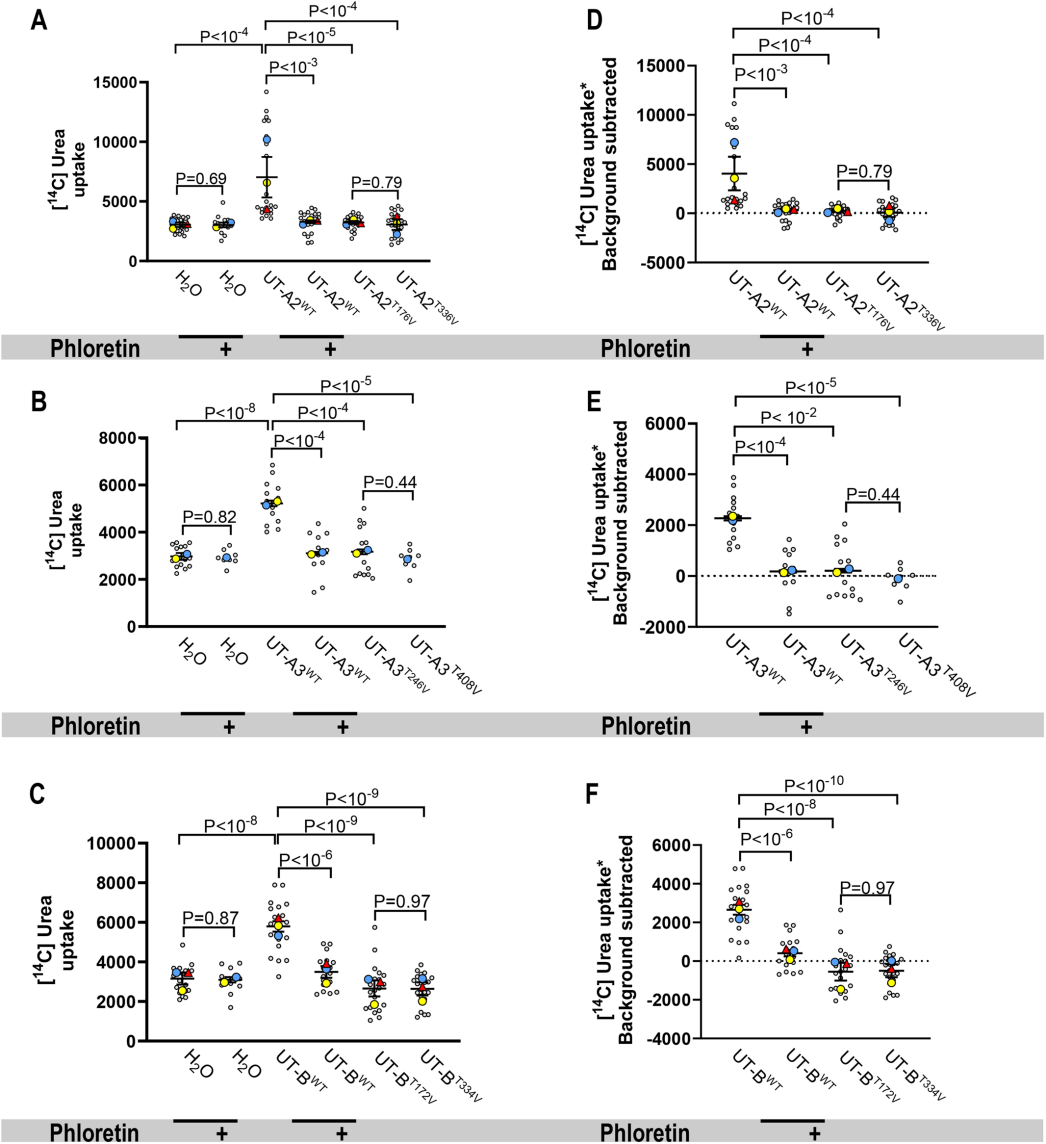
**Summary of the [C^14^] urea uptake of oocytes expressing wild-type or mutant mUT-A2, mUT-A3_,_ or mUT-B.** (A) [^14^C]-urea uptake measurements with water-injected (*n*=25), water injected with phloretin (*n*=14), UT-A2^WT^ (*n*=21)_,_ UT-A2 with phloretin (*n*=21), UT-A2^T176V^ (*n*=21) and UT-A2^T338V^ (*n*=22) oocytes. (B) [^14^C]-urea uptake measurements with water-injected (*n*=15), water-injected with phloretin (*n*=8), UT-A3^WT^ (*n*=13)_,_ UT-A3 with phloretin (*n*=11), UT-A3^T246V^ (*n*=13), and UT-A3^T408V^ (*n*=8) oocytes. (C) [^14^C]-urea uptake measurements with water-injected (*n*=20), water-injected with phloretin (*n*=10), UT-B^WT^ (*n*=19)_,_ UT-B^WT^ with phloretin (*n*=17), UT-B^T172V^ (*n*=21), and UT-B^T334V^ (*n*=22) oocytes. Panels D–F on the right display background-subtracted results that yield channel-dependent activity. Gray circles indicate the values of each oocyte. Colored circles and triangles represent the averages of experiments performed on different days. The horizontal and vertical (error bars) lines on each plot represent the overall mean and the standard error of the mean of each group and condition. A standard two-tailed Student's *t*-test with Bonferroni correction was used to compare the difference between two means, as indicated above each graph. The significance level was set at *P*<0.0125.

The mean ^14^C-urea uptake values were measured for oocytes expressing wild-type or mutant UTs and day-matched water-injected control oocytes (Fig. 3). Oocytes expressing UT-A2^WT^ (Fig. 3A), UT-A3^WT^ (Fig. 3B), or UT-B^WT^ (Fig. 3C) took up significantly more ^14^C-urea than the water-injected control cells. Additional groups of oocytes were incubated with 0.5 mM phloretin, a known UT inhibitor in *Xenopus* (Geyer et al., 2013b; Yang and Verkman, 1998) and *Lithobates* (Kabutomori et al., 2018, 2020) oocytes to verify that the augmented urea uptake was UT-mediated. Indeed, urea uptake by oocytes expressing the wild-type UTs was phloretin-sensitive, attenuating urea uptake to levels that were not significantly different from control oocytes (Fig. 3A–C). Phloretin did not affect urea uptake into water-injected oocytes. Concerning the UT-A2, UT-A3, or UT-B Thr to Val mutations, the urea uptake values of oocytes expressing these constructs were not significantly different from the day-matched water-injected control cells.

Subtracting the mean ^14^C-urea uptake of day-matched water-injected control cells (background) from the ^14^C-urea uptake value for each UT-expressing oocyte provides an estimate of the UT-dependent urea uptake ([^14^C]-urea*) (Geyer et al., 2013b; Kabutomori et al., 2020). The bar graphs in Fig. 3D–F summarize computed oocyte-by-oocyte ^14^C-urea uptake differences for mUT-B^WT^, mUT-A2^WT^, mUT-A3^WT^, and the Thr mutants, respectively. The mean [^14^C]-urea* values show that oocytes expressing mUT-A2^WT^, mUT-A3^WT^, and mUT-B^WT^ exhibit urea uptake significantly greater than zero, and phloretin inhibition or Thr mutation reduced [^14^C]-urea* to values not different from zero. Thus, these conserved Thr residues play an essential role in UT-A-mediated urea transport, as previously reported for bovine and human UT-Bs (Geyer et al., 2013b; Levin et al., 2012).

### Osmotic water permeability

**Fig. 4.**
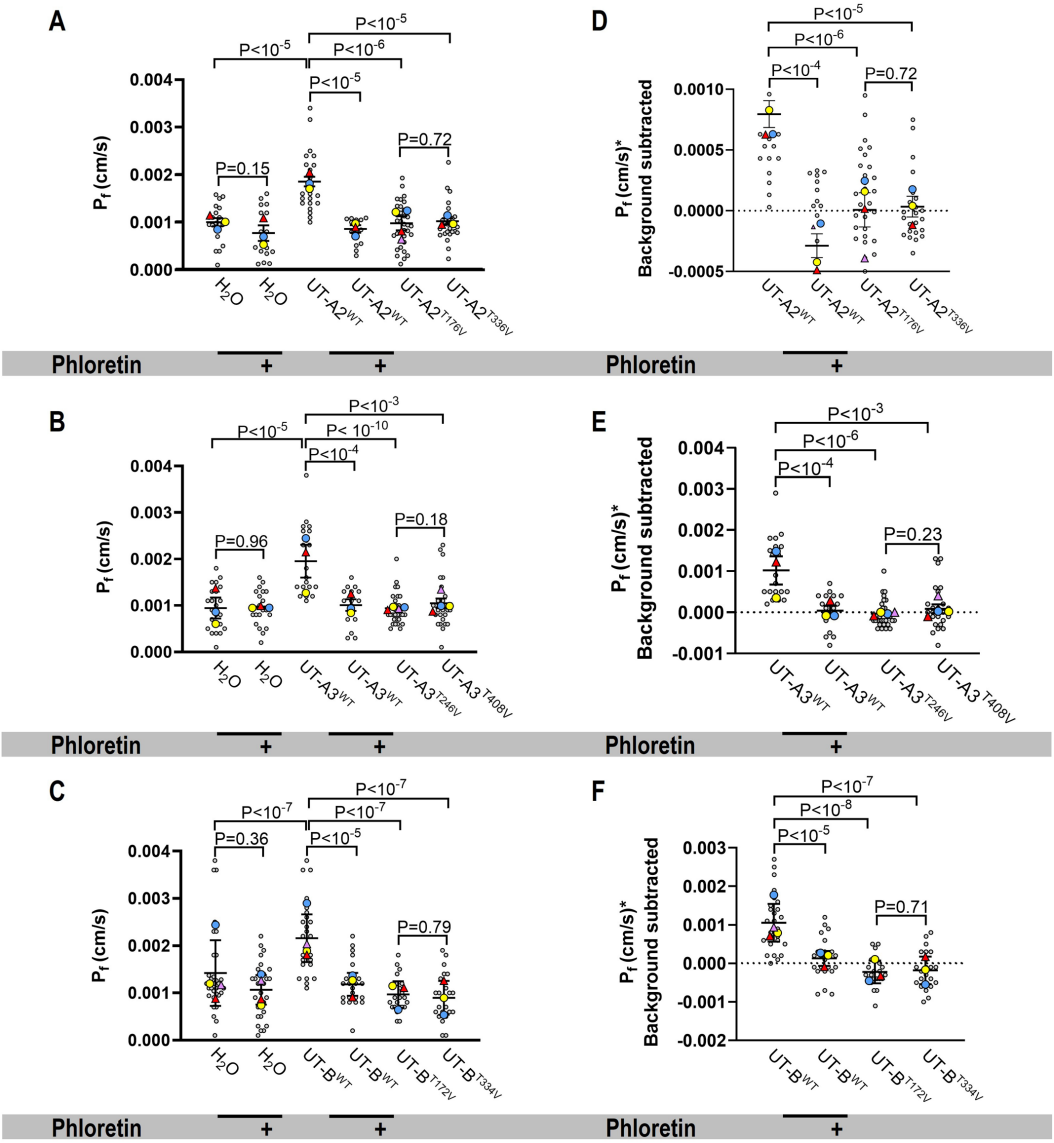
**Summary of osmotic water permeability of oocytes expressing wild-type or mutant mUT-A2, mUT-A3_,_ or mUT-B.** (A) Osmotic water permeability (P*_f_*) measurements with water-injected (*n*=21), water-injected with phloretin (*n*=17), UT-A2^WT^ (*n*=21)_,_ UT-A2 with phloretin (*n*=15), UT-A2^T176V^ (*n*=34), and UT-A2^T338V^ (*n*=26) oocytes. (B) P*_f_* measurements with water-injected (*n*=22), water-injected with phloretin (*n*=20), UT-A3^WT^ (*n*=20)_,_ UT-A3 with phloretin (*n*=17), UT-A3^T246V^ (*n*=30), and UT-A3^T408V^ (*n*=27) oocytes. (C) P*_f_* measurements with water-injected (*n*=27), water-injected with phloretin (*n*=23), UT-B^WT^ (*n*=24)_,_ UT-B^WT^ with phloretin (*n*=21), UT-B^T172V^ (*n*=22), and UT-B^T334V^ (*n*=23) oocytes. Panels D–F on the right display background-subtracted results that yield channel-dependent activity. The P*_f_* was monitored by acquiring one image per second for 100 s. Gray circles indicate the values of each oocyte. Colored circles and triangles represent the averages of experiments performed on different days. The horizontal and vertical (error bars) lines on each plot represent the overall mean and the standard error of the mean of each group and condition. A standard two-tailed Student's *t*-test with Bonferroni correction was used to compare the difference between two means, as indicated above each graph. The significance level was set at *P*<0.0125.

The P*_f_* values of UT-expressing (wild-type and mutants) oocytes were compared with day-matched water-injected control oocytes. The mean P*_f_* values for mUT-A2^WT^ (Fig. 4A), mUT-A3^WT^ (Fig. 4B), and mUT-B^WT^ (Fig. 4C) were significantly greater than the day-matched control cells. Phloretin significantly reduced the P*_f_* of oocytes expressing wild-type UTs but did not affect water-injected oocytes, which displayed a negligible P*_f_*. Like the urea uptake results, oocytes expressing the UTs with Thr to Val mutations had P*_f_* values that were not statistically different from control oocytes.

The mean UT-dependent P*_f_* (P_f_*) values, computed oocyte-by-oocyte, revealed that the P_f_* values for mUT-A2^WT^ (Fig. 4D), mUT-A3^WT^ (Fig. 4E), and mUT-B^WT^ (Fig. 4F) were significantly greater than zero. Phloretin pre-treatment and both Thr mutations decreased P_f_* to values that were not different from zero.

These results confirm that UT-A2 and UT-A3 facilitate water transport and that the Thr residues in the urea pore selectivity filter are necessary for this process, as described previously for human UT-B (Geyer et al., 2013b).

### NH_3_ permeability

**Fig. 5.**
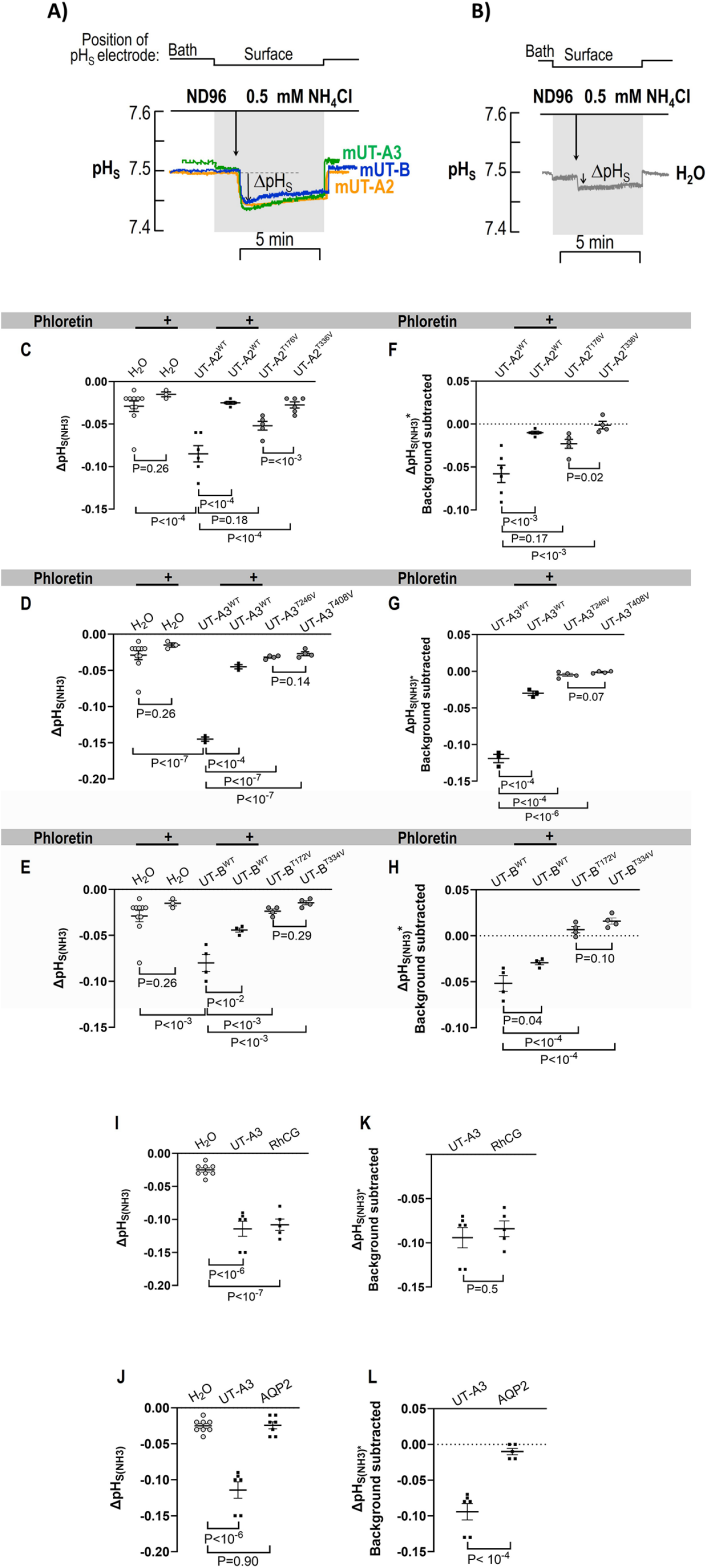
**Summary of changes in surface pH following ammonia exposure of oocytes expressing wild-type or mutant mUT-A2, mUT-A3_,_ or mUT-B.** (A) Changes in surface pH [ΔpH_S(NH3)_] of oocytes expressing wild-type mUT-A2, mUT-A3_,_ or mUT-B following exposure to 0.5 M NH_3_/NH_4_^+^. (B) Changes in surface pH [ΔpH_S(NH3)_] of water-injected oocytes following exposure to 0.5 M NH_3_/NH_4_^+^. (C) Surface pH measurements with water-injected (*n*=10), water-injected with phloretin (*n*=3), UT-A2^WT^ (*n*=6), UT-A2 with phloretin (*n*=5), UT-A2^T176V^ (*n*=5), and UT-A2^T338V^ (*n*=6) oocytes following exposure to 0.5 M NH_3_/NH_4_^+^. (D) Surface pH measurements with water-injected (*n*=10), water-injected with phloretin (*n*=3), UT-A3^WT^ (*n*=3), UT-A3 with phloretin (*n*=3), UT-A3^T246V^ (*n*=4), and UT-A3^T408V^ (*n*=4) oocytes following exposure to 0.5 M NH_3_/NH_4_^+^. (E) Surface pH measurements with water-injected (*n*=10), water-injected with phloretin (*n*=3), UT-B^WT^ (*n*=4), UT-B^WT^ with phloretin (*n*=4), UT-B^T172V^ (*n*=4), and UT-B^T334V^ (*n*=4) oocytes. Panels F–H on the right display background-subtracted results that yield channel-dependent activity. Oocytes expressing RhCG (*n*=5; panels I and K) or AQP2 (*n*=7; panels J and L) were used as positive and negative controls for assessing NH_3_ transport. Gray circles indicate the values of each oocyte. Colored circles and triangles represent the averages of experiments performed on different days. The horizontal and vertical (error bars) lines on each plot represent the overall mean and the standard error of the mean of each group and condition. A standard two-tailed Student's *t*-test with Bonferroni correction was used to compare the difference between two means, as indicated above each graph. The significance level was set at *P*<0.0125.

Previous studies using *Xenopus* oocytes demonstrated that membrane protein-mediated increases in NH_3_ influx can be detected by measuring pH at the cell surface with pH-sensitive glass microelectrodes, as indicated by transient an acidification at the oocyte surface (Musa-Aziz et al., 2009, 2010). As shown in Fig. 5A, positioning the electrode on the surface and switching from ND96 to 0.5 mM NH_4_Cl produces a transient decrease (i.e. acidic) in the ΔpH_S(NH3)_ of oocytes due to UT-mediated base (NH_3_) influx, which drives the reaction NH_4_^+^ --> NH_3_ + H^+^ at the cell surface producing an acidic pHSs transient. In contrast, water-injected oocytes display just a slight change in pH_S_ when the solution is changed (Fig. 5B).

Fig. 5C–E shows that the ΔpH_S(NH3)_ of water-injected control oocytes is negligible and unaffected by phloretin. On the other hand, oocytes expressing mUT-A2^WT^ (Fig. 5C), mUT-A3^WT^ (Fig. 5D), or mUT-B^WT^ (Fig. 5E) exhibited substantial ΔpH_S_(NH_3_) signals, indicating NH_3_ transport. Phloretin treatment significantly reduced ΔpH_S(NH3)_ of oocytes expressing mUT-A2^WT^, mUT-A3^WT^, or mUT-B^WT^. Moreover, the Thr mutations also attenuated the movement of NH_3_ across the oocyte membrane.

Subtracting the average ΔpH_S_ signal of the water-injected oocytes from the ΔpH_S_ signal of the UT-expressing oocytes yields the channel-dependent ΔpH_S(NH3)_ [ΔpH_S(NH3)_^*^]. Fig. 5F–H show significant attenuations in ΔpH_S(NH3)_^*^ following the treatment of oocytes expressing wild-type UTs with phloretin and in oocytes expressing the UTs with the Thr mutations.

As positive and negative controls for the pH_S_ experiments, oocytes injected with cRNA encoding for RhCG or AQP2 were also evaluated. As shown in Fig. 5I, RhCG-expressing oocytes have ΔpH_S(NH3)_ values significantly greater than water-injected oocytes and zero and statistically similar to UT-A3-expressing oocytes (Fig. 5K). In contrast, the AQP2-expressing oocytes' ΔpH_S(NH3)_ values were not significantly different from control cells (Fig. 5J) or zero (Fig. 5L).

These results confirm that the observed ΔpH_S(NH3)_ effects observed with UT-A2 and UT-A3 are due to the expression of these proteins in the oocyte's membrane, which are responsible for the NH_3_ influx. Furthermore, the Thr residues in the urea pore selectivity filter appear to facilitate this process, as previously reported for UT-B (Geyer et al., 2013b).

## DISCUSSION

In the present study, we demonstrated that, in addition to transporting urea and water, UT-A2 and UT-A3 also enhance the permeability of oocyte membranes to NH_3_. Moreover, chemical inhibition with phloretin and site-directed mutagenesis revealed that these UT isoforms conduct NH_3_ through the urea pore utilizing a mechanism that relies on two conserved Thr residues. These results provide insights into the role(s) of UTs – with their permeabilities to urea, water and NH_3_ – and are an important nexus for integrating the excretion of nitrogenous wastes, water, and acid, which is essential for nitrogen balance, urine concentration, and acid–base homeostasis.

The production of NH_3_/NH_4_^+^ occurs in all tissues of the body through various pathways and processes. The renal production of NH_3_/NH_4_^+^ and its excretion are major mechanisms by which the kidneys produce ‘new HCO_3_^−^’ in response to normal or increased acid load, thereby maintaining acid–base balance (Mohiuddin and Khattar, 2024). The steps of NH_3_/NH_4_^+^ secretion in the CD include interstitial NH_3_ entry into the cell across the basolateral membrane and exit across the apical membrane to titrate, in the lumen, the (parallel) secreted H^+^ to form NH_4_^+^. The theory postulated that NH_3_ transport across the CD epithelium occurs by nonionic NH_3_ diffusion through the lipid matrix of the cell membrane. However, two seminal studies revealed that some cell membranes are impermeable to this base [e.g. the apical membrane of gastric gland cells (Waisbren et al., 1994) and the apical membrane of colonic crypts (Singh et al., 1995)]. A subsequent study proposed that the low permeability of these membranes to NH_3_ may be an intrinsic property of membranes exposed to extreme and stressful environments (Cooper et al., 2002), such as in the kidneys, RBCs and liver.

In the OMCD, NH_3_ transport has been mainly ascribed to the vast distribution of RhBG (basolateral) and RhCG (basolateral and apical) proteins (Biver et al., 2008; Eladari et al., 2002; Gruswitz et al., 2010; Verlander et al., 2003; Weiner and Verlander, 2011) and NH_3_-permeable AQPs (Litman et al., 2009). However, in the IMCD, RhBG expression gradually decreases until it becomes undetectable at the papillary tip (Verlander et al., 2003), and RhCG is completely absent. Moreover, CD-specific RhCG KO mice require seven days of acid-loading before exhibiting reduced NH_4_^+^ excretion, acidic urine production and metabolic acidosis (Lee et al., 2014). The same authors also showed that wild-type and haploinsufficient RhCG mice could handle this acidic challenge (Lee et al., 2014; Weiner and Verlander, 2014). These observations raise the question of how NH_3_/NH_4_^+^ is handled in the inner medulla, particularly in the IMCD, where the final steps of urinary concentration occur. Given the localization of UT-A2 in the tDL and UT-A3 in both the apical and basolateral membranes of IMCD principal cells, along with the previously mentioned NH_3_ transport properties of UT-B, this study assessed the NH_3_ transport properties and mechanism of these UT-A isoforms to fill in this gap.

As shown in Fig. 2, all the wild-type and mutant UT cRNAs were translated, and the proteins were post-translationally glycosylated and inserted into the oocyte membrane. As expected, oocytes expressing wild-type mUT-A2, mUT-A3, and mUT-B displayed augmented urea uptake and P*_f_* values. We also demonstrated that UT-A isoforms, like UT-B, can transport NH_3_. It should be pointed out that stopped-flow experiments using vesicles prepared with *Xenopus* oocyte plasma membrane expressing mUT-A2 or mUT-A3 and loaded with the pH-sensitive fluorescent probe 5,6-carboxyfluorescein failed to detect UT-mediated NH_3_ transport (MacIver et al., 2008). However, this method for monitoring pH changes is less accurate and quantitative than our ΔpH_S_ measurement technique that uses an ion-selective liquid-membrane microelectrode – highly sensitive to H^+^ variations – placed directly on the outer surface of the oocyte membrane, where pH_S_ changes occur due to NH_3_ influx. Moreover, artificial liposomes likely do not have the same permeability properties as whole oocytes.

We also performed ΔpH_S_ experiments with oocytes expressing hAQP2 [NH_3_ impermeable (Geyer et al., 2013a)] and RhCG [NH_3_ permeable (Geyer et al., 2013c)] to further validate our results. We found that AQP2-expressing oocytes displayed ΔpH_S_(NH_3_) values similar to control oocytes. On the other hand, RhCG-expressing oocytes displayed increased ΔpH_S_(NH_3_) values, corroborating the NH_3_ transport of this family of proteins in *Lithobates* oocytes. Thus, we can conclude that UT-A2 and UT-A3 can transport NH_3_.

Concerning the transport mechanism, it has been proposed that the UT-A isoforms utilize the same one as UT-B (Levin and Zhou, 2014), but until now, this has not been assessed experimentally. The inhibition of UT-mediated urea, water and NH_3_ transport by phloretin indicates that all three substances traverse the membrane utilizing the urea channel, since this drug intercalates into and physically occludes the S_o_ region of the urea pore. However, this approach does not provide any information about the selectivity filter. Molecular dynamics simulations performed with bUT-B (Geyer et al., 2013b; Levin et al., 2012) revealed that the energy barriers of the selectivity filter of the monomeric urea channel are relatively low in the S_o_ region, rapidly increase approaching the T334 residue of bUT-B in the S_m_ region and remain elevated until reaching the T172 residue, after which the energy barrier levels quickly drop in the S_i_ and are similar to those of the extracellular region. Levin et al. (2012) mutated the residue analogous to Thr339 in bUT-B to Val and found that urea efflux from liposomes was reduced to control levels. Another study demonstrated that Thr334Val in hUT-B blocked urea, water and NH_3_ transport, while the Thr172Val mutation only impaired that of water and NH_3_ (Geyer et al., 2013b).

**Fig. 6.**
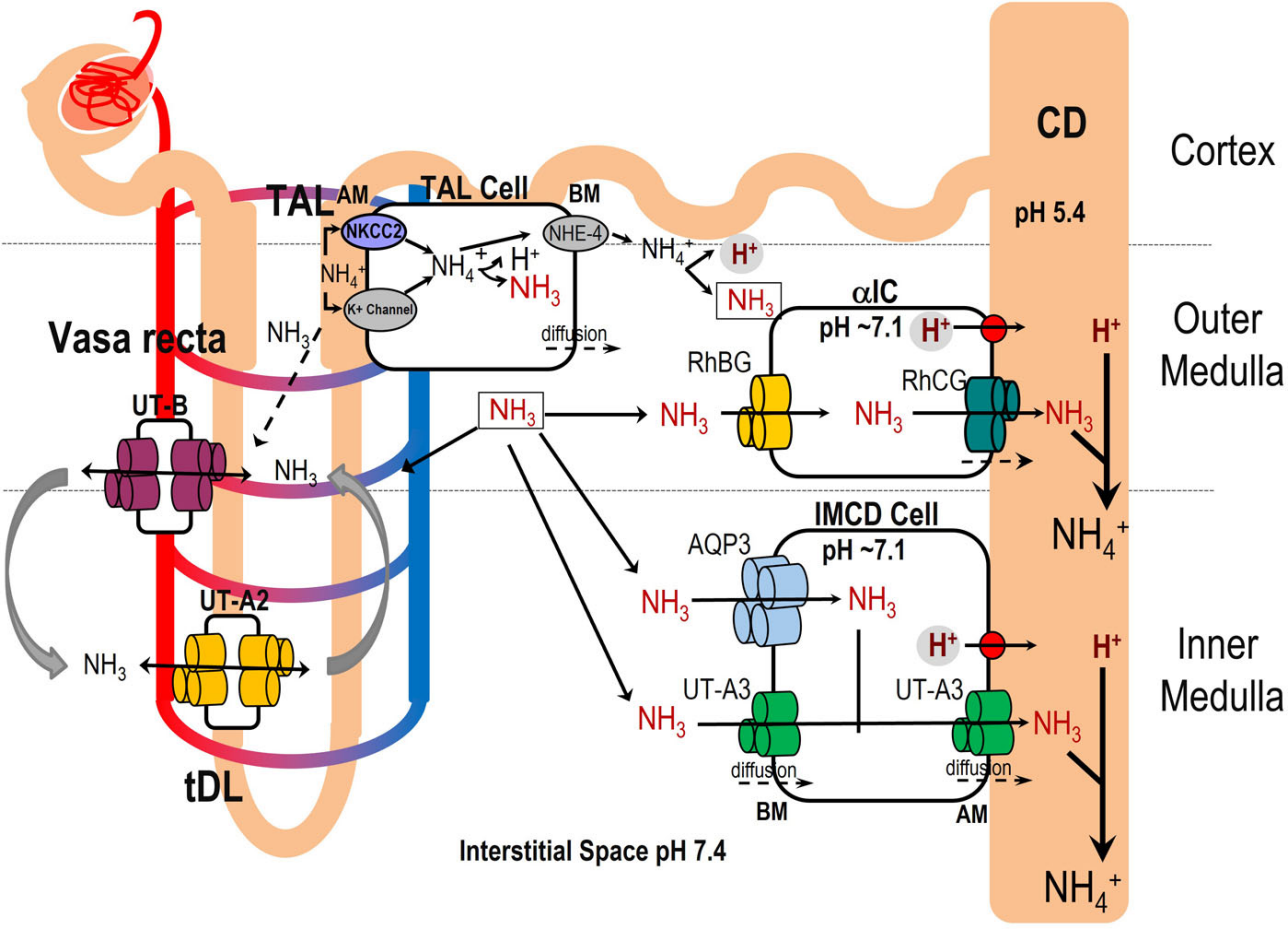
**Model of the potential role of UT-mediated ammonia transport in the renalmedulla.** Ammonium (NH_4_^+^) ions, produced and secreted by proximal tubule (PT) cells, are reabsorbed in the thick ascending limb (TAL) of the loop of Henle via the apical Na^+^-K^+^-2Cl^−^ cotransporters (NKCC2) and, to a lesser extent, K^+^ channels. Inside the TAL cell, NH_4_^+^ is transported across the basolateral membrane to the interstitium via Na^+^/H^+^ exchanger isoform 4 (NHE-4) or dissociates to form NH_3_ and H^+^. The intracellular NH_3_ exits across the TAL basolateral membrane via mechanisms not yet fully elucidated, while the H^+^ exits through the basolateral NHE-4 exchanger. In the medullary interstitium, NH_4_^+^ partially dissociates into NH_3_ and H^+^. Some of this interstitial NH_3_ reenters the thin descending limb (tDL) of the loop of Henle via UT-A2-mediated NH_3_ transport and recycles back to the TAL. A larger portion of the interstitial NH_3_ enters the collecting duct (CD) lumen. In the outer medullary collecting duct (OMCD), RhBG and RhCG on the basolateral side and RhCG on the apical side of the α-intercalated (α-IC) cells transport the NH_3_ into the CD lumen. In the inner medullary collecting duct (IMCD) principal cells, UT-A3 and AQP3 take up NH_3_ on the basolateral side, and UT-A3 on the apical side delivers this base into the CD lumen. H^+^ is secreted into the CD lumen mainly by apical H^+^-ATPase and H^+^-K^+^-ATPase. In the CD lumen, NH_3_ is trapped as NH_4_^+^ and excreted in the urine. A smaller fraction of interstitial NH_3_ is taken up by UT-B in the vasa recta and delivered to the bloodstream. In the blood, the RBCs take up the NH_3_ via AQP1, RhAG and UT-B and deliver it to the liver. AM, apical membrane; BM, basolateral membrane. The solid arrows represent the solute transport. The dashed arrows represent the possible NH_3_ diffusion across plasma membranes.

In the present study, the ΔpH_S_(NH_3_) values of UT-A2^WT^ and the UT-A2^T176V^ mutant were not significantly different. In contrast, despite being expressed on the oocyte surface, the UT-A2^T334V^ mutation significantly attenuated the urea*, P_f_^*^, and ΔpH_S_(NH_3_)^*^ to values not significantly different from zero. Thus, it appears that Thr334 (equivalent to Thr339 in hUT-B) is more critical than Thr176 (equivalent to Thr177 in hUT-B) with regard to solute passage through the selectivity filter of UT-A2. On the other hand, the ΔpH_S_(NH_3_) values of UT-A3^T246V^ and UT-A3^T408V^ were significantly attenuated compared to UT-A3^WT^. Thus, we can confirm that urea, water, and NH_3_ traverse the lipid membrane utilizing the same pathway (i.e. the urea pore) and filtering mechanism previously described for mammalian UT-Bs. From a physiological perspective, this newly ascribed NH_3_ transport function mediated by UT-A2 and UT-A3 could be relevant to effectively maintaining acid–base balance. In Fig. 6, we propose a model describing the potential physiological role of UT-mediated NH_3_ transport along the tubular structures and renal vasa recta.

The regulation of renal ammonium excretion plays a central role in maintaining systemic acid–base balance. Briefly, to maintain the plasma HCO_3_^−^ concentration and thus plasma pH in a normal range, the kidney operating at steady state must reabsorb (reclaim) all the load of HCO_3_^−^ filtered by the glomerulus and generate ‘new HCO_3_^−^’ to replenish the HCO_3_^−^ consumed to neutralize the daily acid load (Weiner and Verlander, 2013). The major pathway responsible for generating ‘new HCO_3_^−^’ is predominantly related to the glutamine metabolism in the PT cells (Good and Burg, 1984; Weiner and Verlander, 2013), which produces equimolar amounts of NH_4_^+^ and HCO_3_^−^ for each glutamine metabolized. The HCO_3_^−^ produced is transported across the basolateral plasma membrane via Na^+^ – HCO_3_^−^ cotransporter NBCe1 (Boron and Boulpaep, 1983; Romero et al., 1997) and delivered to the bloodstream as ‘new HCO_3_^−^’. Importantly, the NH_4_^+^ must be excreted in the urine to prevent these ions from going to the liver, where they would be detoxified by conversion to urea in a process that consumes HCO_3_^−^, yielding no net benefit to acid–base balance (Weiner and Verlander, 2013). Thus, the excretion of NH_4_^+^ in the urine is also an essential task of the kidneys in maintaining the acid–base balance. Indeed, the kidneys increase ammonia excretion substantially in response to exogenous acid loads (Tannen and Sahai, 1990; Weiner and Hamm, 2007). In this sense, the regulation of renal ammonium excretion plays a central role in maintaining systemic acid–base balance.

Interestingly, most renal NH_3_/NH_4_^+^ excretion is due to intrarenal production, not glomerular filtration, in the PT (Good and Burg, 1984). The NH_4_^+^ produced and secreted by PT cells (Nagami, 1989; Weiner and Verlander, 2013) is, as shown in our model in Fig. 6, reabsorbed in the TAL of the loop of Henle mainly via the apical NKCC2 and K^+^ channels when NKCC2 is inhibited (Attmane-Elakeb et al., 2001; Good, 1994; Weiner and Verlander, 2013) with NH_4_^+^ replacing K^+^. Additionally, it has been demonstrated that NH_4_^+^ uptake is augmented across the apical TAL membrane during metabolic acidosis and potassium depletion (Attmane-Elakeb et al., 1998; Good, 1990; Tannen, 1977; Weiner and Hamm, 2007).

Inside the TAL cell, NH_4_^+^ can be transported across the basolateral membrane via Na^+^/H^+^ exchanger isoform 4, [NHE-4, where NH_4_^+^ competes with H^+^ for binding to the H^+^-transporter (Bobulescu and Moe, 2006; Bourgeois et al., 2010)] or it can dissociate to form NH_3_ and H^+^. In the latter case, via mechanisms not yet fully elucidated (Jakobsen et al., 2004; Wang et al., 2020; Weiner and Verlander, 2013) – and beyond the scope of this manuscript – NH_3_ exits across the TAL basolateral membrane and enters the interstitial fluid of the renal medulla, while the intracellular H^+^ exits across the basolateral NHE-4. Regardless of the mechanism, NH_4_^+^ in the medullary interstitium – juxtaposed with the epithelium of the CD due to the anatomical arrangement of the renal medulla – encounters a pH of approximately 7.4, partially dissociating into NH_3_ and H^+^.

Our model proposes that, in the tDL, UT-A2 facilitates the reuptake of a portion of the NH_3_ from the medullary interstitium and recycles it back to the TAL of the loop of Henle lumen. A previous study reported that NH_3_ recycling is largely driven by NH_3_ rather than NH_4_^+^ transport (Flessner et al., 1993). This process could produce an axial medullary interstitium NH_3_ gradient to maintain medullary acid–base homeostasis (Good et al., 1987; Weiner and Verlander, 2013). The larger portion of the interstitial NH_3_ enters the CD – later excreted in the urine as NH_4_ – while the smaller portion, transported by UT-B in the vasa recta, enters the bloodstream and is delivered to the liver, which converts NH_4_^+^ into urea (Weiner et al., 2015). The RBCs can take the NH_3_ up through UT-B and NH_3_-permeable RhAG (Geyer et al., 2013c) and AQP1 (Geyer et al., 2013b).

In the medullary interstitium, at pH 7.4, the NH_3_/NH_4_^+^ ratio, according to the Henderson–Hasselbalch equation, is 1/63. In the CD lumen, where pH can reach 5.4 (two pH units lower than the pH of the medullary interstitium), the NH_3_/NH_4_^+^ ratio is 1/6300. Thus, there is a favorable gradient for the transport of NH_3_ from the interstitium across the basolateral and apical membranes to the CD lumen, which increases during acidosis (Weiner et al., 2015). Indeed, several studies have reported evidence that NH_4_^+^ secretion into the CD involves parallel secretion of H^+^ and NH_3_ into the lumen without significant transepithelial transport of NH_4_^+^ (Bakouh et al., 2006; DuBose et al., 1991; Geyer et al., 2013c; Knepper, 1991; Weiner and Verlander, 2011).

However, in the CD lumen, sustained NH_4_^+^ formation requires secretion of both NH_3_ and H^+^. Geyer et al. (2013c) proposed that H^+^ in the interstitium reacts with the HCO_3_^−^ [transported through the anion exchanger AE1 (Romero, 2005) in the basolateral membrane of the α-intercalated (α-IC) cells of the CD], producing CO_2_ and H_2_O, under the catalytic action of GPI-anchored CAIV in the basolateral membrane (Purkerson and Schwartz, 2007). The CO_2_ enters the CD cell across the basolateral membrane and combines with H_2_O, producing H^+^ and HCO_3_^−^ mediated by the catalytic action of CAII (Purkerson and Schwartz, 2007). Thus, the HCO_3_^−^ source entering the cell through the basolateral membrane is CO_2_ (Geyer et al., 2013b). Inside the cell, the HCO_3_^−^ ions are recycled back into the medullary interstitium through basolateral AE1, and the H^+^ is secreted into the CD lumen.

It is well-known that the H^+^ ions in the CD cell are primarily secreted into the CD lumen by apical H^+^-ATPase and H^+^-K^+^-ATPase in the apical membrane of the α-IC cells of the CD system (Gumz et al., 2010; Valles et al., 2006). In the OMCD, the membrane glycoproteins RhCG (apical membrane) and RhBG (basolateral membrane) have been shown to facilitate NH_3_ transport into the CD lumen, thereby being critical for NH_4_^+^ excretion in response to metabolic acidosis (Bishop et al., 2010; Eladari et al., 2002; Gruswitz et al., 2010; Lee et al., 2009, 2010, 2014; Wagner et al., 2011; Weiner and Verlander, 2011). On the other hand, besides AQP3 in the basolateral membrane of IMCD principal cells, no other known ammonia transporters have been identified (Geyer et al., 2013a). As shown in the proposed model in Fig. 6, our results suggest that the interstitial NH_3_ could also be taken up by UT-A3 on the basolateral membrane of IMCD principal cells and delivered to the CD lumen by UT-A3 on the apical membrane. The low pH environment in the CD lumen (pH 5.4) promotes a rapid reaction between NH_3_ and H^+^ ions, reforming NH_4_^+^, which is then excreted in the urine.

Furthermore, the fraction of NH_3_ returned to systemic circulation could be taken up by UT-A2 (Sands and Blount, 2014) and UT-B in the liver (Sands, 2000), facilitating detoxification via the urea cycle by conversion into urea and glutamine (Weiner et al., 2015). In this case, the newly formed urea could then be transported out of the liver cells – again through UT-A2 and UT-B – taken up by the RBCs via UT-B and delivered to the kidney for excretion. This scenario highlights how using a single protein capable of transporting the substrates and products of a chemical reaction (i.e. NH_3_, H_2_O, and urea) could improve the catalytic efficiency and lower the cell's energetic demands.

It should be noted that attempts to heterologously express full-length UT-A1, which is also expressed in the apical membranes of IMCD cells, in *Lithobates* oocytes were unsuccessful. While it is tempting to speculate that the full-length UT-A1 protein displays the same transport properties as each half, it is understood that urea and water transport in the CD need to be regulated separately. Previous studies have shown that members of the AQP protein family transport a variety of solutes, including urea, glycerol, hydrogen peroxide, ions, as well as CO_2_ and NH_3_. For example, the CO_2_ permeability of AQP4 changes depending on the oligomerization state. In this case, full-length AQP4_M1_ is impermeable to CO_2_, and the AQP4_M23_, which is missing the first 22 amino acids and forms orthogonal arrays of particles, is permeable to this dissolved gas (Geyer et al., 2013a). Additionally, AQP2, which is expressed in the apical membranes of IMCD cells, only transports water despite substantial sequence and structural similarities to the NH_3_-permeable AQPs. Furthermore, as shown in Fig. 1C and D, UT-A1 cannot form trimers, like UT-B or UT-A3, meaning that it remains monomeric or forms dimers, which could impact the protein's transport properties. Thus, like AQP2 being specific for water transport in the apical membrane of the IMCD, UT-A1 could be urea-specific. This possibility also offers a potential explanation for why UT-A1 and UT-A3 are both expressed in IMCD cells. In this sense, the presence of UT-A3 on the apical and basolateral membranes could afford additional or compensatory urea, water and NH_3_ transport. Future studies, perhaps using different cell types, will need to be conducted to evaluate this possibility.

As reviewed by Fenton (2008), knocking out UT-A1, UT-A3, or UT-B, individually, in combinations, or globally, profoundly affects urinary concentration due to impaired urea accumulation in the inner medulla. On the other hand, UT-A2 KO mice displayed urinary concentration function similar to wild-type animals but showed decreased urine osmolality when fed a low-protein diet and deprived of water, demonstrating the role of this transporter under urea-limited conditions (Fenton, 2008; Uchida et al., 2005). Unfortunately, none of the studies reported the blood and/or urine pH of these animals. It would not be surprising if these parameters were unaltered in the KOs because the three families of NH_3_ transporters (i.e. UTs, Rhs, and AQPs) are often colocalized in areas of the body actively involved in NH_3_/NH_4_^+^ detoxification and excretion, such as the kidneys, liver and RBCs (Litman et al., 2009). Therefore, knocking out more than one NH_3_ transporter family may be necessary to reveal any substantial physiological consequences. This observation highlights the importance of NH_3_/NH_4_^+^ handling since compensatory mechanisms have been established to maintain acid–base balance when other transport pathways are compromised, or the organism is overwhelmed.

While there is no direct evidence for the involvement of UT-A-mediated NH_3_ transport in health and disease, there is some evidence at the level of genome-wide studies (GWAS) that indicates associations between conditions or traits and *SLC14A2* variants (https://www.ebi.ac.uk/gwas/genes/SLC14A2). For example, in addition to systolic and diastolic blood pressure disturbances, altered urea, creatine and blood urea nitrogen (BUN) levels and estimated glomerular filtration rate were reported. UT-A-mediated urea and NH_3_ transport could contribute to the altered urea and BUN levels. In contrast, none of the traits or conditions of the *SLC14A1* variants (https://www.ebi.ac.uk/gwas/genes/SLC14A1) could be attributed to impaired or augmented NH_3_ concentrations. On the contrary, many variants were associated with altered hematological parameters, which is not surprising given the high abundance of UT-B in the RBCs (Azouzi et al., 2013). Future studies, *in vitro* and/or *in vivo*, evaluating the gene and protein expression levels of these proteins in specific renal cells/compartments under different experimental conditions (i.e. dehydration, metabolic acidosis, and diet) need to be conducted to verify these observations.

Regarding therapeutic potential, based on the impaired urinary concentration phenotype in the whole animal and targeted UT KO models, small molecule inhibitors against these transporters have emerged as salt-sparing diuretics (i.e. urearetics) (Klein and Sands, 2016). These drugs could promote urine output when traditional diuretics for fluid overload in heart and liver failure patients do not work (Anderson et al., 2012; Yao et al., 2012). There is evidence that nonspecific (inhibition of UT-A≈UT-B) inhibitors induce diuretic activity (Li et al., 2020). While there are currently no UT-A-specific inhibitors, it has been proposed that they would directly increase fractional urea excretion by blocking IMCD urea uptake (Titko et al., 2020) and produce fewer adverse effects than UT-B inhibitors (Nandi et al., 2021). As illustrated in Fig. 1, designing UT-A1 inhibitors is further complicated by the presence of two independent urea pores (i.e. UT-A2 and UT-A3), in other words, two targets. In this sense, it is conceivable that a drug could bind to and inhibit only half of UT-A1 (UT-A2 or UT-A3), never achieving complete inhibition.

With regard to the UT-B inhibitors that have been developed, in addition to being an effective diuretic by increasing urine output, the UT-B-specific inhibitor UTB_inh_-14 may also have therapeutic potential for treating neuroinflammation (Jones et al., 2021). It has been shown that UTB_inh_-14's selectivity for UT-B is likely due to Leucine 116 (human numbering), which is an Alanine in UT-A isoforms (Anderson et al., 2012; Yao et al., 2012) and an extracellular drug-binding pocket that is not present on the more negatively charged UT-A surface (Chi et al., 2023). Based on our UT-mediated NH_3_ transport results, the effects of UT inhibitors on renal NH_3_/NH_4_^+^ handling and consequently on acid–base homeostasis should be considered, since blocking some of the NH_3_ pathways in the inner medulla may have physiological consequences.

### Conclusion

In addition to transporting urea and water, we demonstrated that mUT-A2 and mUT-A3 facilitate the transmembrane movement of NH_3_. Chemical inhibition and site-directed mutagenesis revealed that the three substances all pass through the urea channel and rely on two Thr residues in the selectivity filter of the pore. UT-mediated renal NH_3_ transport could contribute to establishing NH_3_ gradients in the inner medulla necessary for NH_3_ secretion. This process is critical for NH_4_^+^ excretion and, consequently, for maintaining acid–base balance, especially during an acidosis.

## MATERIALS AND METHODS

The complete details of the *L. catesbeianus* oocyte heterologous expression system were described in two manuscripts from our laboratory (Kabutomori et al., 2018, 2020). This system was developed to circumvent the obstacles to obtaining and utilizing *Xenopus laevis* oocytes in countries like Brazil, which has strict restrictions on importing certain animal species.

### Plasmids

We used pP7TS plasmids harboring the wild-type mUT-B (AF448798), mUT-A2 (AF367359) and mUT-A3 (AF258602) genes, with C-terminal c-Myc tags (Kabutomori et al., 2020; MacIver et al., 2008). Wild-type hAQP2 in the pP7TS expression vector (Geyer et al., 2013a) and hRhCG in the BSXG expression vector (Brown et al., 2009; Geyer et al., 2013c) were used as positive and negative controls in the functional assays and co-expression experiments.

### Site-directed mutagenesis

The amino acid residues corresponding to Thr177 and Thr339 in hUT-B (i.e. Thr176 and Thr338 in mUT-A2, Thr246 and Thr408 in mUT-A3, and Thr172 and Thr334 in mUT-B) were individually mutated to Valines using the QuikChange II Site-Directed Mutagenesis Kit (Agilent Technologies, Santa Clara, CA, USA). The mutagenic primers were designed using the QuikChange Primer Design Program (www.chem.agilent.com/store/primerDesignProgram.jsp) and synthesized by Exxtend Solução in Oligos (São Paulo, Brazil). The forward and reverse primer pairs to generate the mUT-A2^T176V^ and mUT-A2^T338V^, mUT-A3^T246V^ and mUT-A3^T408V^, and mUT-B^T172V^ and mUT-B^T334V^ mutations are listed in Table 1.

**Table 1.** Reverse and forward primers for each mutation

Construct	Primers
UT-B^T172V^	Forward 5′→3′ GCCATGTTGAAAGGGAGAACAAAGACGGGCAGGTCCCACTT Reverse 5′→3′ AAGTGGGACCTGCCCGTCTTTGTTCTCCCTTTCAACATGGC
UT-B^T334V^	Forward 5′→3′GCTGTGGTTCACCTGCCAGCTTGTGTGTGGTCCTTTTGTTTG Reverse 5′→3′CAAACAAAAGGACCACACACAAGCTGGCAGGTGAACCACAGC
UT-A2^T176V^	Forward 5′→3′ GTGGGACCTCCCAGTCTTCGTACTGCCCTTCAACATC Reverse 5′→3′ GATGTTGAAGGGCAGTACGAAGACTGGGAGGTCCCAC
UT-A2^T338V^	Forward 5′→3′ TTGGATTACCACCCTGCGTCTGGCCCTTTTGCCTC Reverse 5′→3′ GAGGCAAAAGGGCCAGACGCAGGGTGGTAATCCAA
UT-A3^T246V^	Forward 5′→3′ GGGACCTGCCCGTCTTCGTCCTGCCCTTCAACATTGC Reverse 5′→3′ GCAATGTTGAAGGGCAGGACGAAGACGGGCAGGTCCC
UT-A3^T408V^	Forward 5′→3′ TTGGGGTGCCCTCAGGCGTTTGGGCCTTCTGTCTCTC Reverse 5′→3′ GAGAGACAGAAGGCCCAAACGCCTGAGGGCACCCCAA

Mutations are highlighted in grey.

Following the manufacturer's protocol, the PCR-based mutation reactions contained 50 ng of wild-type UT cDNA template, 125 ng of each forward and reverse primer, 1 µl of 10 mM dNTP stock, 1 µl of DNA polymerase (2.5 U/µl) and ddH_2_O to a final volume of 20 μl in a 200 µl PCR tube. After gently mixing the reaction mixtures, the samples were transferred to a Veriti 96-well Thermal Cycler (Applied Biosystems, Foster City, CA, USA). The cycling program included one denaturation cycle at 95°C for 30 s, followed by 18 amplification cycles with denaturing at 95°C for 30 s, annealing at 55°C for 1 min, and extending at 68°C for 4.5 min. At the end of the run, samples were maintained at 4°C. Ten units of *Dpn*I restriction enzyme were added to the PCR samples, and the tubes were incubated in a 37°C water bath overnight to digest away the methylated parental DNA.

The next day, using the heat shock method, 1 µl of the *Dpn*I-digested sample was used to transform supercompetent XL1-Blue cells (Agilent Technologies). The transformed cells were spread onto LB-ampicillin (100 μg/ml) plates and incubated overnight at 37°C. Individual colonies were selected to inoculate 5 ml of LB-ampicillin (100 μg/ml) media. These cultures were grown overnight at 37°C with shaking at 250 rpm. The plasmid DNA of the entire culture was isolated and purified using a DNA Miniprep Kit (Qiagen, Valencia, CA, USA) and sequenced.

### DNA sequencing

All purified cDNAs were sequenced in the forward and reverse directions using the BigDye Terminator v3.1 Cycle Sequencing Kit (Applied Biosystems) and an ABI Prism 3130XL Genetic Analyzer (Hitachi, Tokyo, Japan). All the nucleotide and translated protein sequences of the wild-type and mutant mUTs, hAQP2, and hRhCG were confirmed.

### cRNA synthesis

Sequence-verified UT-encoding cDNAs were linearized with *Xba*I (New England Biolabs, Ipswich, MA, USA) and purified using the QIAquick PCR Purification Kit (Qiagen). *Eco*RI (New England Biolabs) was used to linearize the pT7TS plasmid containing hAQP2, and *Xho*I restriction enzyme (New England Biolabs) was used to linearize the pBSXG plasmid harboring human RhCG (hRhCG). The linearized DNAs were transcribed into capped RNAs (cRNAs) using the T7 mMachine Kit (Ambion, Austin, TX, USA). The synthesized cRNAs were purified with the RNeasy MinElute RNA Cleanup Kit (Qiagen). The concentration and purity of all DNAs and RNAs were quantified by measuring the absorbance at 260 and 280 nm with a Nanodrop 2000c spectrophotometer (Thermo Fisher Scientific, Waltham, MA, USA).

### Heterologous expression in *Lithobates* oocytes

#### Animals

The Committee of Animal Care and Use at the Institute of Biomedical Sciences of the University of São Paulo approved all surgical and experimental procedures involving animals (protocol #7971160519). Adult female *Lithobates* frogs were purchased from ‘Rã’s’ World (São Paulo, Brazil) and maintained in an aquatic tank at 22°C, with a protein-based diet (Poli-Nutri, São Paulo, Brazil) and a 12-h light: dark cycle. The frogs that underwent the ovariectomy surgery weighed 350–450 g.

#### Surgery

Frogs were anesthetized by immersion with 0.2% ethyl 3-aminobenzoate methanesulfonate (Tricaine, Sigma-Aldrich) in 5 mM HEPES, pH 7.50. A 1.0–1.5 cm incision was made lateral to the abdominal midline, and ovarian fragments were surgically removed.

#### *Lithobates* oocyte isolation

The ovary fragments were digested with 0.25 mg/ml collagenase type VII (Sigma-Aldrich) in a 0-Ca^2+^ solution (82 mM NaCl, 2 mM KCl, 20 mM MgCl_2_, 5 mM HEPES, pH 7.45) for 5 min at room temperature to isolate the oocytes. Robust stage V–VI oocytes were manually selected and mechanically defolliculated. The oocytes were transferred to OR3 media [6.85 g/l of powdered Leibovitz L-15 media supplemented with L-glutamine and 100 ml of 10,000 U/ml penicillin and 10,000 mg/ml streptomycin (Gibco, Grand Island, NY, USA) and 5 mM HEPES] and stored at 18°C until microinjection.

#### cRNA microinjection

The cRNA injection needles were pulled with a Sutter Instrument Co. Model P-97 Flaming/Brown micropipette puller (Novato, CA, USA) and aseptically cut to produce a tip approximately 2 μm in diameter (Musa-Aziz et al., 2010). The needles were filled with mineral oil and placed on a Nanoliter 2000 microinjector (World Precision Instruments, Sarasota, FL, USA).

Individual oocytes were injected with 25 nl of 1 µg/µl mUT-A2^WT^_,_ mUT-A2^T176V^, mUT-A2^T338V^, mUT-A3^WT^_,_ mUT-A3^T246V^, mUT-A3^T408V^, mUT-B^WT^_,_ mUT-B^T172V^, mUT-B^T334V^, hAQP2, or hRhCG cRNA or the same volume of sterile water (i.e. day-matched water-injected control cells). Following injection, oocytes were stored in OR3 media at 18°C for 3–4 days until processing and analysis. The OR3 media was changed daily, and dead cells were removed to avoid contamination.

### Surface expression assessment

#### Biotinylation

Membrane protein expression patterns of UT- and water**-**injected oocytes were assessed using the EZ**-**Link Sulfo**-**NHS**-**Biotinylation Kit (Thermo Fisher Scientific), followed by immunoblotting, as previously described for *Xenopus* (Geyer et al., 2013a, b,c) and *Lithobates* (Kabutomori et al., 2018, 2020) oocytes. For each independent experiment, 20 UT-injected and 20 water-injected oocytes were evaluated.

Following surface biotinylation, the oocytes were homogenized by repeatedly pipetting them up and down through a P200 pipette tip. The samples were centrifuged at 3000× ***g*** for 10 min at 4°C, and the supernatants (∼200 µl) were collected and transferred to new 1.5 ml Eppendorf tubes. For each sample, 20 µl of the sample extract [i.e. total protein extract (i.e. intracellular+surface)] were removed and stored on ice. The remaining 180 µl of the supernatant was mixed with an equal volume of NeutrAvidin (Thermo Fisher Scientific) and incubated in a sealed Spin X column (Corning, Pittston, PA, USA). After the incubation and washing steps, 180 µl of elution buffer (1× sample buffer plus 0.5 M DTT) were added to the spin columns and incubated for 1 h at room temperature on a rocker platform. The biotinylated proteins were eluted into sterile collection tubes by centrifugation at 1000× ***g*** for 1 min.

#### Immunoblots

Total and surface samples from wild-type and mutant UTs and water-injected day-matched control oocytes were separated on 12% Tris-glycine SDS-PAGE gels and transferred to PVDF membranes. The membranes were blocked with Tris-buffered saline (TBS) containing 0.1% Tween-20 (TBST) and 5% powdered milk (TBST-B) for 1 h at room temperature. The membranes were incubated with a monoclonal primary anti-c-Myc antibody (cat. #1849372, Invitrogen, Carlsbad, CA, USA) overnight at 4°C. The next day, the blots were washed three times with TBS (10 min each) and incubated with a secondary goat anti-mouse antibody conjugated to horseradish peroxidase (HRP) (cat. #041806, KPL, Gaithersburg, MD, USA) for 1 h at room temperature. The Pierce ECL Plus Substrate (Thermo Fisher Scientific) was used to visualize the immunoreactive bands. Images were captured with an Amersham Imager 600 (GE Healthcare Life Sciences, Logan, Piscataway, NJ, USA).

Experiments using oocytes injected with hAQP2 or hRhCG employed the same processing and detection protocols used for the UTs, except that a polyclonal anti-AQP2 antibody (Alpha Diagnostics, San Antonio, TX, USA) or a polyclonal antibody raised against the C-terminal region of RhCG (Brown et al., 2009) and a goat anti-rabbit secondary antibody conjugated to HRP (AP132P; Millipore, Billerica, MA, USA) were used as reported previously (Geyer et al., 2013a,c).

### Physiological measurements

#### Urea uptake

Urea uptake was evaluated by monitoring [^14^C]-urea transport into oocytes (Geyer et al., 2013b). Groups of five oocytes (UT-injected or water-injected) were placed in 200 μl of ND96 (96 mM NaCl, 2 mM KCl, 1 mM MgCl_2_, 1.8 mM CaCl_2_, and 5 mM HEPES) containing 5 μCi of [^14^C]-urea (PerkinElmer, Waltham, MA, USA) plus 1 mM of unlabeled urea. After 10 min, the oocytes were washed in ND96 with 1 mM of unlabeled urea. Each oocyte group was lysed in 100 μl of 5% SDS in water by pipetting the samples up and down through a P200 pipette tip. The lysed oocytes were transferred to a scintillation container containing 5 ml of scintillation fluid for [C^14^] analysis. Oocytes were pre-incubated in ND96 plus 0.5 mM phloretin (Sigma-Aldrich) for 20 min before the urea uptake assay to evaluate inhibition (Kabutomori et al., 2020).

#### Osmotic water permeability

Osmotic water permeability (P*_f_*) was determined using video microscopy to monitor cell swelling following exposure to a hypotonic solution (Geyer et al., 2013a; Kabutomori et al., 2018, 2020; Musa-Aziz et al., 2009). Groups of six oocytes were placed in the hypotonic ND96 solution variant (70 mosmol/L H_2_O), and cell swelling was monitored using a Nikon SMZ 745T stereoscopic microscope (Melville, NY, USA) connected to a digital camera (Optix Cam, Roanoke, VA, USA). A 1.3 mm diameter metallic ball bearing was placed in the field as an internal reference. One image per second was acquired for 100 s. The P*_f_* (cm/s) was calculated by measuring each oocyte's projection area over time using the ImageJ software (NIH, Bethesda, MD, USA). For inhibition experiments, oocytes were pre-incubated in ND96 plus 0.5 mM of phloretin for 20 min before the assay (Geyer et al., 2013b).

#### Surface pH measurements

This surface pH (pH_S_) technique was initially developed and utilized to monitor membrane protein-mediated NH_3_ and CO_2_ transport across *Xenopus* oocyte membranes (Musa-Aziz et al., 2009, 2010). pHs was measured using a liquid-membrane H^+^-selective microelectrode (20 μm tip diameter) filled with H^+^ ionophore mixture B (cat. #95293, Fluka) and connected to an FD223 electrometer (World Precision Instruments, WPI). The external reference electrode was a calomel half-cell, bridged to the chamber via a glass micropipette filled with 3M KCl (amplified by a model 750 electrometer, WPI). pH_S_ was calculated by subtracting the reference signal from the pH electrode signal.

Before starting the pHs measurements, an oocyte was placed and secured in a perfusion chamber (channel dimensions: 3 × 30 mm) and continuously perfused with ND96 solution (pH 7.50) at a rate of 3 mL/min. Perfusion was controlled by syringe pumps (Harvard Apparatus, South Natick, MA), and the solutions were changed using pneumatically operated valves. All experiments were performed at room temperature (∼22 °C). In the chamber, the pHs microelectrode was calibrated in ND96 solution (pH 7.50) before applying the 0.5 mM NH_4_Cl solution (pH 7.50). Using an ultra-fine micromanipulator (MPC-200 system, Sutter Instrument Company, Novato, CA, USA), the flat tip of the pHs electrode was positioned at the oocyte surface and then gently advanced ∼40 μm to create a dimple in the membrane. This depression creates a microenvironment between the electrode tip and the oocyte surface, where the chemical reactions occur and can be detected by the pHs electrode. During the experiment, the pHs electrode was withdrawn 300 μm for recalibration in the extracellular solution (pH 7.50). We measured the maximum pHs transient (ΔpHs) after switching the extracellular solution from ND96 (pH 7.50) to 0.5 mM NH_4_Cl (pH 7.50). This change leads to NH_3_ influx into the oocyte, lowering NH_3_ concentration near the surface and driving the reaction NH_4_^+^ → NH_3_ + H^+^ to occur. The released H^+^ from this reaction causes an acidic pHs transient that is detected by the pHs microelectrode. The maximum decay of pHs (ΔpH_S_) is a semiquantitative index of the NH_3_ influx (Musa-Aziz et al., 2009).

Oocytes expressing hAQP2 [water permeable/NH_3_ impermeable (Geyer et al., 2013a)] were used as negative and positive controls for NH_3_ and water transport, and oocytes expressing hRhCG [NH_3_ permeable/water impermeable (Geyer et al., 2013c)] were used as positive and negative controls for NH_3_ and water transport. For inhibition experiments, oocytes were pre-incubated in ND96 plus 0.5 mM of phloretin for 20 min before the assay (Geyer et al., 2013b).

### Statistics

Experiments performed on different days were grouped separately. The mean was calculated for each replicate and used to determine the overall mean and the standard error of the mean (s.e.m.). Standard two-tailed Student's *t*-tests were performed to compare the difference between two means, and the significance level was adjusted to *P*<0.0125 using the Bonferroni correction. The averages were represented by colored symbols (circles and triangles), while gray circles indicate the values of individual oocytes. Statistical analyses were performed using the GraphPad Prism Software v.10.3.0 (GraphPad Software, Inc., San Diego, CA, USA).
